# Germinal center architecture disturbance during *Plasmodium berghei *ANKA infection in CBA mice

**DOI:** 10.1186/1475-2875-6-59

**Published:** 2007-05-16

**Authors:** Leonardo JM Carvalho, Maria F Ferreira-da-Cruz, Claudio T Daniel-Ribeiro, Marcelo Pelajo-Machado, Henrique L Lenzi

**Affiliations:** 1 Laboratory of Malaria Research, Department of Immunology, Instituto Oswaldo Cruz, FIOCRUZ, Rio de Janeiro, Brasil; 2 Department of Pathology, Instituto Oswaldo Cruz, FIOCRUZ, Rio de Janeiro, Brasil

## Abstract

**Background:**

Immune responses to malaria blood stage infection are in general defective, with the need for long-term exposure to the parasite to achieve immunity, and with the development of immunopathology states such as cerebral malaria in many cases. One of the potential reasons for the difficulty in developing protective immunity is the poor development of memory responses. In this paper, the potential association of cellular reactivity in lymphoid organs (spleen, lymph nodes and Peyer's patches) with immunity and pathology was evaluated during *Plasmodium berghei *ANKA infection in CBA mice.

**Methods:**

CBA mice were infected with 1 × 10^6 ^*P. berghei *ANKA-parasitized erythrocytes and killed on days 3, 6–8 and 10 of infection. The spleen, lymph nodes and Peyer's patches were collected, fixed in Carson's formalin, cut in 5 μm sections, mounted in glass slides, stained with Lennert's Giemsa and haematoxylin-eosin and analysed with bright-field microscopy.

**Results:**

Early (day 3) strong activation of T cells in secondary lymphoid organs was observed and, on days 6–8 of infection, there was overwhelming activation of B cells, with loss of conventional germinal center architecture, intense centroblast activation, proliferation and apoptosis but little differentiation to centrocytes. In the spleen, the marginal zone disappeared and the limits between the disorganized germinal center and the red pulp were blurred. Intense plasmacytogenesis was observed in the T cell zone.

**Conclusion:**

The observed alterations, especially the germinal center architecture disturbance (GCAD) with poor centrocyte differentiation, suggest that B cell responses during *P. berghei *ANKA infection in mice are defective, with potential impact on B cell memory responses.

## Background

Malaria remains one of the major public health problems in the developing world, with an estimate of 300–500 million cases and 1–3 millions deaths every year. The development of a vaccine has been one of the research strategies to face this threat, especially with the spreading of parasite resistance to many of the drugs currently available to treat the disease. However, despite over 30 antigens having been identified as vaccine candidates and many of them having been tested in pre-clinical and up to phase III clinical trials, none of them has so far generated a solid perspective for a vaccine to be available in the coming years [1]. One of the reasons malaria vaccine development has been hindered is the fact that the immune responses and the mechanisms responsible for acquisition of immunity to malaria are largely unknown. Acquisition of immunity to malaria in highly endemic areas such as sub-Saharan Africa is considered to be a slow process requiring many years to take place. Exposed children below five years of age acquire protection against severe manifestations of the disease, remaining susceptible to infection and milder morbidity. As age increases, the frequency of clinical attacks decreases and after puberty most individuals (except pregnant women) present a complete immunity against clinical manifestations of the disease. Yet, most individuals remain susceptible to infection, but the parasite load is greatly decreased and very low parasitaemia are prevalent in adulthood [2,3]. In addition, it seems that this partial, non-sterile, immunity is rapidly lost if the contact with the parasite is discontinued. Many factors seem to contribute to poor immunity in malaria. Among them, it is widely believed that blood stages of the parasite induce immunosuppression and impair the development of immunological memory. This has been questioned by Struik and Riley [4], who argued that some paradigms, especially the lack of memory in the immune response to the malaria parasite, do not have a solid scientific evidence and may be misleading.

In mice, both cellular and humoral responses play important roles in the immunity against blood stage malaria infection [5]. But besides being involved in protection, immune responses in malaria can also trigger immunopathology [6]. In fact, complications such as cerebral malaria and severe anaemia have a strong immunological component in humans [7], as well as in experimental models [8-10]. The *Plasmodium berghei *ANKA infection of CBA mouse is an established model of malaria with neurological involvement (the so-called experimental cerebral malaria), and infection with blood stage parasites leads to 100% lethality. The immune response in this model is not only ineffective against parasite growth, but also responsible for the 60–80% incidence of CM, mainly characterized by strong Th1 T cell responses [11], macrophage hyperactivation [12], and also CD8+ T cell cytotoxicity [13].

Given these characteristics of the immune responses during blood stage malaria, the understanding of the mechanisms leading to poor immunity and immunopathology is crucial for the rational development of prophylactic and therapeutic interventions, such as vaccines. Although immune responses to malaria and physiopathogenesis of cerebral malaria in mice have been widely studied [14-17], detailed analysis of changes in lymphoid compartments, which is basic to understand how the immune system respond to a challenge, has not been widely approached, with a few works mainly on the *Plasmodium chabaudi *model [18-20]. It has recently been shown that *P. berghei *ANKA-infected CBA mice develop various degrees of thymocyte apoptosis, leading in some cases to thymocyte depletion and severe thymus cortical atrophy [21]. In the present work, attention was focused on the sequential immunohistopathological changes caused by *P. berghei *ANKA infection in several lymphoid compartments and their possible implications in pathology and immunity of the infection.

## Materials and methods

### Animals, parasite and infection

Four to 8 week-old female CBA mice (CECAL/Fiocruz, Rio de Janeiro) were inoculated by intraperitoneal injection with 1 × 10^6 ^*P. berghei *ANKA-parasitized erythrocytes obtained from a donor infected CBA mouse. A total of 71 mice in three separate experiments were infected and lymphoid organs of 32 mice were examined by histology, on the following days after infection: three (ten mice); six (two mice); seven (eight mice); eight (two mice); and 10 (ten mice). Seventeen uninfected control mice (age and sex-matched) were examined on days: three (six mice); seven (six mice); and 10 (five mice). Mice were anesthetized with ketamine plus midazolan and bled. Organs were removed immediately after death and transferred to Carson's formalin-Millonig [22]. Organs from mice that died spontaneously were not collected. Protocols were reviewed and approved by the Fiocruz Ethical Committee on Animal Experimentation (CEUA protocol number P-0155-03).

### Parasitaemia and clinical parameters

Density and maturation of circulating parasites were daily determined by examination of Giemsa-stained blood smears collected from the tail vein. Clinical CM was defined by the presentation of at least one of the following signs: limb paralysis, convulsions, stupor and roll over.

### Histopathologic study

Spleen, inguinal and periaortic lymph nodes and Peyer's patches were fixed in Carson's formalin-Millonig [22]. After fixation, the spleen was cut in four pieces in transversal sections, mounted in a box together with four lymph nodes and four Peyer's patches and embedded in paraffin. Sections (5 μm) were stained with haematoxylin-eosin and Lennert's Giemsa [23], and analyzed by bright-field microscopy. Lennert's Giemsa is the best stain to characterize, by bright-field, the cellular populations in lymphoid organs because stain RNA and DNA in blue (allowing to infer the presence of ergastoplasm and the characteristic of nucleoli), acidophilic substances in red by the eosin (discriminates eosinophils) and acid mucopolisaccharides in red-violet (labels mast cell granules).

## Results

**Figure 1:**
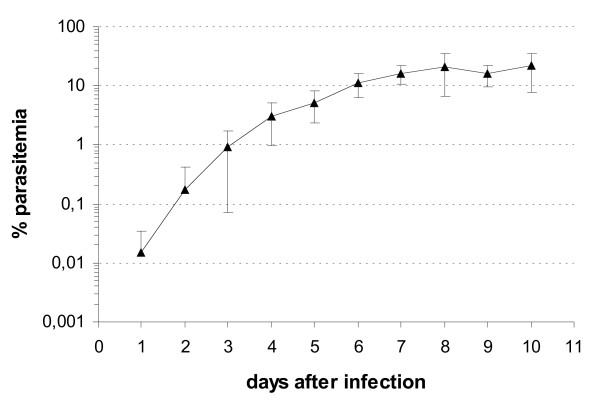
Course of parasitaemia (mean ± 2 standard deviations of 3 separate experiments) in CBA mice inoculated with 1 × 10^6 ^*P. berghei *ANKA-parasitized erythrocytes.

The overall characteristics of infection, including plasma TNF levels and histopathological changes in brain, liver and lungs in mice have been described elsewhere [12]. The course of parasitaemia was as expected, with a rapid growth in the first days, reaching over 10% on day 6 of infection (Figure 1), when the first mice developed CM. Indeed, inoculation of *P. berghei *ANKA to CBA mice resulted in a lethal infection. Ten out of 71 infected mice were examined on day 3 for early histopathological changes. Among the remaining 61 mice, 36 died spontaneously after displaying clinical signs of CM. Twelve mice were examined on days 6 (two mice), 7 (eight mice) and 8 (two mice); although half presented clinical signs of CM (two mice in each day), all twelve mice had some histopathological evidence of brain lesions, including parenchimal, perivascular and/or sub-arachnoid microhaemorrhages, perivascular oedema with few extravasated mononuclear cells, vascular plugging by pigment-containing monocytes and monocyte adherence to endothelial cells. Brain haemorrhages were more prominent in mice with clinical signs of CM (number of haemorrhages/μm^2 ^of brain area: median of 0.65 in CM, and of 0.16 in non-CM mice; mean size of haemorrhages: 3,922 ± 3,061 μm^2 ^in CM and 1,776 ± 658 μm^2 ^in non-CM mice). Vascular plugging was similar in both groups. The thirteen mice that remained did not develop clinical CM and were examined on day 10 (10 mice) or died spontaneously on day 16 (three mice). All infected mice killed presented progressive splenomegaly, detected by measuring spleen/body weight ratio: *i*) day 3: mean: 0,0053 ± 0,0013 (controls: 0,0033 ± 0,0003); *ii*) days 6–8: mean: 0,0097 ± 0,0013 (controls: 0,0033 ± 0,0005); *iii*) day 10: mean: 0,0188 ± 0,0045 (controls: 0,0036 ± 0,0004).

### Histopathology of the spleen

**Figure 2:**
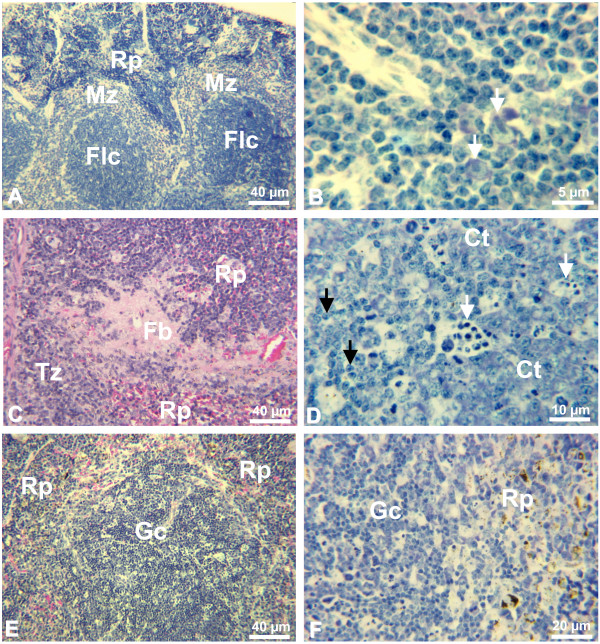
**Spleen**. A) Panoramic view of non-infected control mouse spleen. White and red pulps (Rp) with well-defined limits. Two B cell resting follicles (Flc) with surrounding thick marginal zones (Mz). Giemsa. B) T cell area (periarteriolar lymphoid sheath) of infected mouse (day 3). Immunoblasts (arrows). Giemsa. C) Infected mouse (day 3). Deposit of fibrinoid material (Fb) between T zone (Tz) and red pulp (Rp). Haematoxylin-eosin. D) Infected mouse (day 6). Disorganized germinal center with intense centroblast (Ct) activation and proliferation and apoptosis (white arrows), without centrocyte differentiation. Small lymphocytes (black arrows) permeating the disorganized germinal center. Giemsa. E) Panoramic view of infected mouse spleen (day 6). Disorganized germinal center (Gc), without definition of light and dark areas, absent marginal zone and blurred limits between white and red pulps (Rp). Haematoxylin-eosin. F) Infected mouse (day 6). Blurred limits in the border between disorganized germinal center (Gc) and red pulp (Rp), absent marginal zone, and malarial pigment in red pulp macrophages. Giemsa.

**Figure 3:**
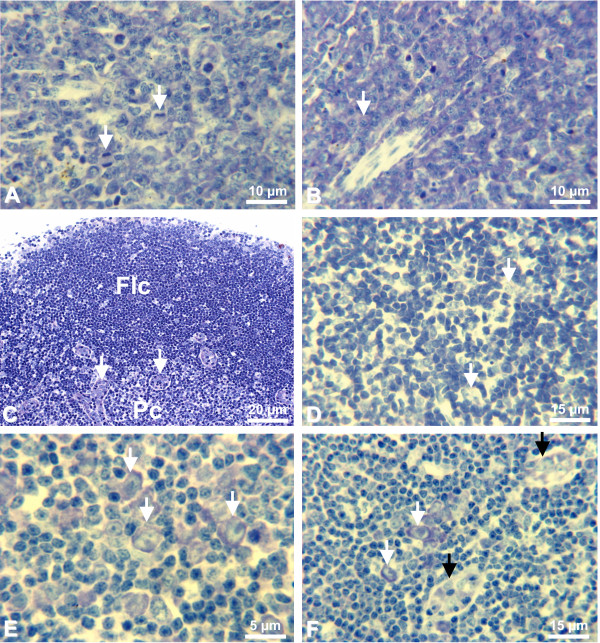
**Spleen/Lymph nodes**. A) Spleen T cell zone of infected mouse (day 6). Intense plasmacytogenesis with mitotic figures (arrows). Giemsa. B) Spleen T cell zone of infected mouse (day 10). Intense plasmacytogenesis at a more advanced stage, with plasma cell (arrow) differentiation. Giemsa. C) Lymph node of non-infected control mouse. Panoramic view of the paracortical (Pc) and follicular (Flc) areas. Giemsa. Arrows: high endothelial venules with trafficking lymphocytes. D) Detail of lymph node paracortical area of non-infected control mouse, showing interdigitating cells (arrows). Giemsa. E) Detail of lymph node paracortical area of infected mouse (day 3). Many immunoblasts (arrows). Giemsa. F) Lymph node of infected mouse (day 3). Immunoblasts (white arrows) and high endothelial venules (black arrows) showing trafficking lymphocytes. Giemsa.

Spleen of non-infected control mice presented quiescent T and B cell zones in the white pulp, resting B-cell follicles, with small lymphocytes, surrounded by prominent marginal zone (Figure 2A). On day 3, several immunoblasts appeared in the periarteriolar lymphoid sheath (T cell region) (Figure 2B). Immunoblasts were also present nearby the marginal zone, which was decreased in size in relation to spleens of non-infected control mice. In the bright-field microscopy, immunoblasts have large light nuclei with very large nucleoli. The latter are often solitary and are found in the middle of the nucleus or at an indentation of the nuclear membrane. The rim of cytoplasm is moderately broad to broad and strongly basophilic, with Giemsa staining [23]. Some foci of plasmacytogenesis were established in the splenic cords. Monocytes with malarial pigment were seen in the marginal zone and in the sinusoids. These alterations were observed in all 10 infected animals examined. One mouse presented a deposit of fibrinoid material in between the T cell zone and the red pulp (Figure 2C). On days 6–8, few immunoblasts were detected in the T zone. Follicle germinal centers (GC) lost the typical architecture acquiring a disorganized aspect (Figure 2D), consisting of intense centroblast proliferation with little centrocytic transformation, several phagocytic-centers with apoptotic bodies, and several perifollicular mitosis. Intense centroblastic proliferation combined with little centrocytic differentiation and centripetal penetration of small lymphocytes from the periphery resulted in loss of definition between dark and light zones and the borders between the GC and the lymphocytes of the mantle zone became indefinable (Figure 2E). Centroblasts and centrocytes were considered according to Lennert's morphological characteristics [23]. Thus, centroblasts (germinoblasts) vary in size and have a round nucleus, the rim of cytoplasm is narrow and strong basophilic. The nuclear chromatin is fine and dispersed. Several medium-size nucleoli are often found at the inner nuclear membrane. Frequently, there are vacuoles in the cytoplasm. Otherwise, centrocytes (germinocytes) are small or medium-sized and conspicuous chiefly because of their notched, often cleaved or irregularly shaped nuclei. The nuclei reveal fine chromatin and very small nucleoli, which are usually central, but occasionally located at the nuclear membrane. The cytoplasm is weak gray-blue with Giemsa staining. Centrocytes can be distinguished from centroblasts by their nuclear form and weak basophilia. They differ from lymphocytes of the mantle zone in their light nucleus, i.e., the nucleus is poor in heterochromatin [23]. The mantle and marginal zones disappeared and the limits between the red pulp and the B zone were blurred (Figure 2E–F). The B zone, in more advanced stage, presented no clear limits with foci of plasmacytogenesis close to follicles. Intense plasmacytogenesis was also observed in the T zone's periarteriolar sheath (Figure 3A). The red pulp was enlarged and contained large number of macrophages with malarial pigment, intense erythropoiesis and foci of monocytopoiesis close to septae. Erythropoiesis was defined by the presence of erythroblast in different stages of maturation and the monocytopoiesis foci were asserted by the occurrence of monoblasts and promonocytes. These changes were observed in all 12 mice analyzed on days 6–8, irrespective of the development of clinical signs of CM. On day 10, the B zone was better defined, persisting with incipient centrocytic differentiation. Plasmacytogenesis was intense in the periarteriolar sheaths and near the follicles, maturing to plasma cell differentiation (Figure 3B). Erythropoiesis was greatly enhanced and there was large quantity of pigment-containing monocytes in the sinusoids.

### Histopathology of the lymph nodes

**Figure 4:**
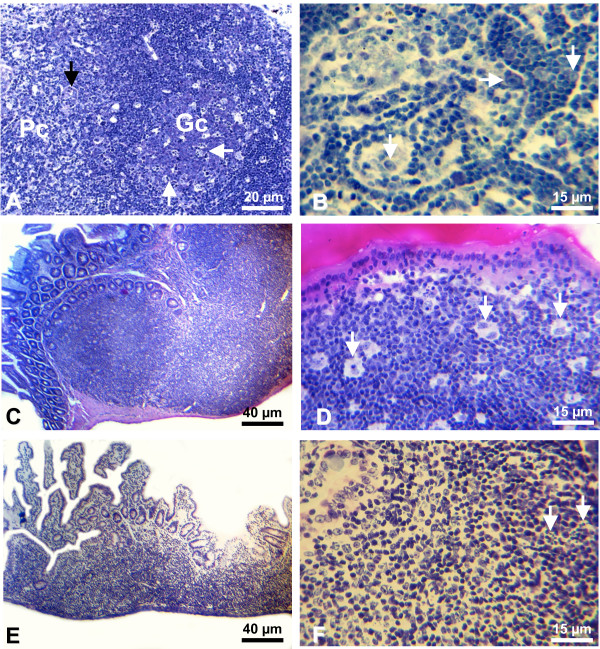
**Lymph nodes/Peyer's patches**. A) lymph node of infected mouse (day 7). Disorganized germinal center (Gc), with small lymphocytes permeating an area of centroblastic proliferation, with several apoptotic bodies (white arrows). Paracortical area (Pc) with high endothelial venules (black arrows). Giemsa. B) lymph node of infected mouse (day 7). Medullary cords showing increased numbers of trafficking small lymphocytes and some immunoblasts (arrows). Giemsa. C) Panoramic view of Peyer patches of non-infected control mouse. Giemsa. D) Peyer patch of non-infected control mouse. Macrophages (arrows) permeating small lymphocytes. Haematoxylin-eosin. E) Panoramic view of Peyer patches of infected mouse (day 7), showing shrinkage of the patch. Giemsa. F) Peyer patch of infected mouse (day 7). Lymphocyte depletion and apoptosis foci (arrows). Haematoxylin-eosin.

Lymph nodes from non-infected control mice were quiescent with resting paracortical area and follicles (Figure 3C–D). All mice killed on day 3 presented a large number of immunoblasts in the paracortical region (Figure 3E–F), and the medullary cords exhibited incipient plasmacytogenesis. On days 6–8, the frequency of immunoblasts in the paracortex had largely decreased, as well as the lymphocyte population in general, with an increase of the interdigitating cells. B-cell follicles presented disorganized GCs, with undefined limits, intense activation and proliferation of centroblasts and some immunoblasts, few centrocytes and penetrating small lymphocytes; lymphocytic corona was absent and small cells spread touching the marginal sinus (Figure 4A). Phagocytic centers with apoptotic bodies were observed in the follicles. On day 10, the paracortical area presented prominence of interdigitating cells. The medullary sinuses were full of lymphocytes and some immunoblasts (Figure 4B). Plasmacytogenesis was intense with more differentiation to plasma cells.

### Histopathology of the Peyer's patches

Non-infected control mice presented quiescent Peyer's patches, with resting small lymphocytes, prominent dome region and several dispersed macrophages (Figure 4C–D). Overall, the alterations during infections were similar to those observed in spleen white pulp and lymph nodes, with many immunoblasts on day 3, intense centroblast activation, proliferation and apoptosis in disorganized GCs on days 6–8. In addition, there was a great depletion of lymphocytes in the dome region on days 6–8, turning the dome monocytes confluent and more evident, resulting in shrunk patches (Figure 4E–F). This histological finding is in accordance with the atrophy of the Peyer's patches observed macroscopically.

## Discussion

*P. berghei *ANKA infection in CBA mice leads to CM and multiorgan pathology, and much of its physiopathogenesis is owed to players of the immune system, with critical involvement of cytokines, T lymphocytes, macrophages and other mediators. The activation of the immune system in this model, whose intensity leads to the pathological consequences, is however largely ineffective to halt parasite growth and even mice that do not develop CM eventually die of hyperparasitaemia. This study shows that *P. berghei *ANKA infection in CBA mice leads to striking changes in secondary lymphoid organs.

Early (day 3) in the infection, there was a strong T cell response in lymph nodes, spleen and Peyer's patches, evidenced by the presence of many immunoblasts in the T cell areas of these organs. CM pathology is dependent on T cells, mainly CD4+ cells [24], outlining the importance of this early widespread activation of T cells. The most remarkable changes were coincident with the time of CM occurrence, on days 6–8 of infection, consisting mainly of striking activation and proliferation of B cells accompanied by GC architecture disturbance (from now called GCAD). Although polyclonal B cell activation and proliferation lead to high immunoglobulin production in malaria [25], and a possible role for immunoglobulins in the expression of human CM has been reported [26], it is unlikely that the atypical GC reaction plays a role in CM pathogenesis in this murine model, since it has been shown that B cell-knockout mice develop CM [27]. Indeed, the GCAD was observed in mice with and without clinical signs of CM on days 6–8, and also in those killed on day 10, which did not develop CM.

The major defect in the GCAD seems to be B cell differentiation (centrocyte transformation), not activation and proliferation (centroblast reaction), which, instead, are overwhelming. In the *P. chabaudi *model, the presence of Peanut Agglutinin (PNA) positive cells in spleen follicles of infected mice has been considered evidence that a normal GC reaction occurs during infection [20]. However, PNA staining does not differentiate cell types (centroblasts, centrocytes), so it cannot detect the poor cell differentiation as it was detected here by conventional Giemsa staining.

The defect in centrocyte transformation is a phenomenon of utmost importance. The GC is a tightly controlled microenvironment for B cell expansion, differentiation or death [28-30]. Centrocyte transformation is related to affinity-maturation of B cells and to the switch of immunoglobulin isotype. B cells that undergo somatic mutation in the Ig variable region genes and show improved affinity for the antigen are rescued from death at this stage through cooperation with follicular dendritic cells and become centrocytes. If the somatic mutation leads to lower affinity to antigen, the B cell cannot receive the rescue signals from follicular dendritic cells and die by apoptosis. Centrocytes then represent cells that ultimately may become the IgG-producing plasma cells when further stimulated. And more, they will be responsible for the secondary response to reinfection, playing a central role in the immunological memory. Struik and Riley [4] have made a critical and elegant review of the immunological memory in malaria, challenging the widespread belief that memory response to malaria is defective. However, the data presented here, although derived from a murine model, strengthen the notion that memory B cell response during acute plasmodial infection must in fact be affected, and deficient centrocyte transformation may then be one of the reasons for the difficulty in developing effective immune responses after repeated malaria infections. Therefore, overcoming this deficiency must be crucial for developing immunoprophylactic interventions such as vaccines.

Another aspect of the B cell response in GCAD is the occurrence of intense apoptosis. Some papers describe that apoptosis is increased during malarial infections in humans [31,32] and in mice [18,19]. By using flow cytometry, Helmby and coworkers [19] showed that the vast majority of apoptotic cells in the spleen of *P. chabaudi*-infected mouse were B cells, consistent with the present histological data. Different interpretations are given to this event, such as a physiological feedback mechanism to control the number of cells after clonal expansion and also an escape mechanism through which the parasite would cause immune unresponsiveness by causing lymphocyte death. Given the GCAD, apoptosis was interpreted as a consequence of the defective centrocyte transformation. Since proliferation is intense and differentiation with affinity maturation is affected, intense apoptosis should indeed be expected to occur as a consequence.

Although centrocyte transformation seems to be impaired, plasmacytogenesis is overwhelming. Plasmablasts could be derived from the marginal zone B cells, known to respond rapidly to blood-borne antigens due to their location adjacent to the marginal sinuses and their proximity to antigen-trapping macrophages and dendritic cells [30]. Following activation, marginal zone B cells become plasmablasts and exit the marginal zone, undergoing a burst of proliferation and forming plasma cell foci in extrafollicular regions such as the periarteriolar lymphoid sheath [33], as in the present case. This could help explaining the disappearance of the marginal zone on days 6–8 of infection. This possibility has been proposed by others, who identified cells with phenotypic characteristics of marginal zone B cells spread throughout the spleen during the acute phase of the *P. chabaudi *infection [20]. It is important to point out that marginal zone B cells do not represent postgerminal center T-dependent B cells and proliferate much faster in response to very low doses of polyclonal mitogens and terminally differentiate, without the participation of germinal center, into plasma cells within hours of activation [34].

Some works have addressed the lymphoid reactions in malaria infections, most data coming from the *P. chabaudi *model and focused in spleen changes. Sanchez-Torres and coworkers [18] have described the enlargement and disarray of the white pulp during acute *P. chabaudi *infection in CB6F1 mice. The disarray was characterized by the lack of definition of the limits between white and red pulps and, at the time of greater number of apoptotic cells, they described an almost complete disappearance of the red pulp. In that model, mice spontaneously recover of infection and white pulp reacquired organization three weeks after the control of parasitaemia. Although that work did not go into detail on the description of the specific white pulp compartments where the disarray occurred, it is likely that this event may be similar to that observed in the *P. berghei *ANKA model. However, in the latter, the red pulp was enlarged, contrary to what was described for the *P. chabaudi *model.

Urban and coworkers [35] have performed an integrated histological and immunochemistry analysis of spleens from patients that died of severe *P. falciparum *malaria, including cases of cerebral malaria. They describe, among other alterations, the cell depletion in the marginal zone, the lack of GC formation and a decrease in the number of B cells in the spleen. The authors interpret the lack of GCs and overall disorganization in spleen architecture as playing possible roles in the poor memory responses in malaria. Similar conclusions were reached in the present work but, as discussed above, based in different data and interpretations. First, in the murine model, GC formation is not absent, it is indeed remarkable, but it is highly disturbed, particularly the B cell differentiation. Second, in contrast to the data of Urban and coworkers, B cell numbers are not decreased, actually follicular B cell proliferation and plasmacytogenesis (GC dependent and/or independent?) are overwhelming and, despite the red pulp enlargement, B cell expansion seems to be the main responsible for the observed splenomegaly. These differences may be real, due to differences in the lymphoid reactions in humans and in the mouse, but can most probably be related to the method of study. Urban and coworkers based their definition of lymphoid compartments and cell types on HE staining and immunophenotyping (in the case of B cells, by using an anti-CD20 monoclonal antibody). In the present work, findings were based on morphology revealed by Giemsa staining. Both approaches present limitations. Immunohistochemistry can provide a good definition of the distribution of different cell types. However, since this is based on the detection of specific cell markers by monoclonal antibodies, subpopulations that may change the expression of a given marker during differentiation can become undetectable depending on the monoclonal antibody used. This is a main difficulty in characterizing mature B cell populations, because changes in morphological characteristics due to activation, proliferation and differentiation are accompanied by phenotypic changes in cell surface and cytoplasmic markers, such as IgD, IgM, IgG, CD19, CD20, B220 and others. For instance, CD20 is not uniformly expressed in the mature B cell lineage, being expressed in naïve and activated B cells but not in plasmablasts or plasma cells [36], and it is then possible that these cells can be missed in immunohistochemistry studies based on this marker. A panel of monoclonal antibodies is then necessary to overcome this kind of limitation. Similarly, conventional histological stainings such as Giemsa, although providing definition of cell types with a high degree of confidence based on differential morphology, do not guarantee 100% certainty. To clarify this point and further characterize the phenomenon, different and complementary techniques (Giemsa-based analysis associated to immunochemistry, for instance) performed in tissues of the same human or animal samples can be envisaged.

The phenomena and the interpretations described in this paper deserve further investigation in order to help unraveling the mechanisms leading to pathogenesis or poor immunity in malaria. The use of other host-parasite combinations and further characterization of cell types can provide a better understanding of the phenomena described here. This is important to allow improved therapeutic interventions and rational designs of effective vaccines.

## Authors' contributions

LJMC conceived the study and carried out the experimental work, read the slides, interpreted results and wrote the manuscript. MFFC and CTDR participated in the study design, data discussion and critically reviewed the manuscript. MPM and HLL participated in the experimental work, read the slides, interpreted results and critically reviewed the manuscript.
